# Cognitive behavioral therapy for a woman with depression and systemic lupus erythematosus

**DOI:** 10.1192/j.eurpsy.2023.2176

**Published:** 2023-07-19

**Authors:** F. Znaidi, S. Ellini, W. Cherif, C. Mejda, D. Rahma

**Affiliations:** 1 Psychiatry department “Ibn Omrane”, Razi Hospital of Manouba, Manouba, Tunisia

## Abstract

**Introduction:**

Cognitive Behavioral Therapy (CBT) provide a means of improving mental health among people with depression. However, few studies have explored its effectiveness with the presence of comorbid somatic disorders.

**Objectives:**

We aimed throug a case report to describe the cognitive and behavioral management of a patient with depression comorbid with systemic lupus erythematosus.

**Methods:**

We present the case of a 47-year-old woman followed since 2012 for a systemic lupus erythematosus. In september 2013, she was diagnosed with depression. The cognitive behavioural therapy took place in 12 sessions of 45 minutes each, one session per week. Initial and final evaluations included Beck’s Depression Inventory and the « Questionnaire des pensées automatiques ».

**Results:**

During the course of the therapy, we noticed an improvement of the patient’s mood, a decrease in anhedonia and somatic complains. We also observed a decrease in instinctual disorders. The final evaluation showed a significant improvement of the different scales. The objectives set with the patient were achieved.

**Conclusions:**

Cognitive behavioral therapy is an interesting option for the management of cases of depression, including its comorbid form with a disabling disease such as systemic lupus erythematosus.

**Disclosure of Interest:**

None Declared

